# The Children’s Picture Books Lexicon (CPB-Lex): A large-scale lexical database from children’s picture books

**DOI:** 10.3758/s13428-023-02198-y

**Published:** 2023-08-11

**Authors:** Clarence Green, Kathleen Keogh, He Sun, Beth O’Brien

**Affiliations:** 1 Faculty of Education, University of Hong Kong, Pok Fu Lam, Hong Kong; 2 Senior Lecturer, Centre for Smart Analytics & Institute of Innovation, Science and Sustainability, Federation University Australia, Mount Helen, Australia; 3 Centre for Research in Child Language, National Institute of Education, Nanyang Technological University, Singapore, Singapore; 4 Centre for Research in Child Development, National Institute of Education, Nanyang Technological University, Singapore, Singapore

**Keywords:** Lexical database, Child input norms, Picture books, Early print exposure, Age of acquisition

## Abstract

This article presents cpb-lex, a large-scale database of lexical statistics derived from children’s picture books (age range 0–8 years). Such a database is essential for research in psychology, education and computational modelling, where rich details on the vocabulary of early print exposure are required. Cpb-lex was built through an innovative method of computationally extracting lexical information from automatic speech-to-text captions and subtitle tracks generated from social media channels dedicated to reading picture books aloud. It consists of approximately 25,585 types (wordforms) and their frequency norms (raw and Zipf-transformed), a lexicon of bigrams (two-word sequences and their transitional probabilities) and a document-term matrix (which shows the importance of each word in the corpus in each book). Several immediate contributions of cpb-lex to behavioural science research are reported, including that the new cpb-lex frequency norms strongly predict age of acquisition and outperform comparable child-input lexical databases. The database allows researchers and practitioners to extract lexical statistics for high-frequency words which can be used to develop word lists. The paper concludes with an investigation of how cpb-lex can be used to extend recent modelling research on the lexical diversity children receive from picture books in addition to child-directed speech. Our model shows that the vocabulary input from a relatively small number of picture books can dramatically enrich vocabulary exposure from child-directed speech and potentially assist children with vocabulary input deficits. The database is freely available from the Open Science Framework repository: https://tinyurl.com/4este73c.

## Introduction

There is a significant need for a lexical database derived from a large sample of children’s picture books. Such a database is essential for research questions in psychology, education and computational modelling, which require rich details on the vocabulary in early print exposure. This paper reports on the development and release of the Children’s Picture Book Lexicon (cpb-lex), the largest such lexical database yet created for English. The resource leverages computational methods to extract speech-to-text closed captions generated from social media videos of presenters reading aloud children's picture books. A picture book is a type of book that relies on pictures and words to tell a narrative and is aimed at young children (0–8 years) (Matulka, 2008; Moya-Guijarro, 2016). The media from which data were extracted reflect shared book reading, during which an adult reads text aloud and children listen and view the illustrations and print (Evans et al., 2008). Picture books are a central text type in learning to read and are used to connect a child’s oral language to the print-based literacy required in school. Such texts are used internationally in formal English medium education (Sun et al., 2020; Wasik et al., 2016) and play an important, albeit variable, role in the home lives of children and parent–child interactions (Heath, 1982; Logan et al., 2019; O’Brien et al., 2020; Sun et al., 2016; Sun & Ng, 2021).

The vocabulary of picture books is a rich source of language input (Dawson et al., 2021). Being read to, joint reading and independent reading improve social, cognitive and educational outcomes for a child (Green & McLachlan, 2023; Krashen, 2004). Literacy ‘gaps’, such as those that exist between socio-economic groups, or between children who are native English speakers and children for whom English is an additional language (EAL), have been cited as economic, social and public health issues (Erbeli & Rice, 2022). Hart and Risley (2003) describe vocabulary input differences between higher and lower socio-economic classes as a ‘catastrophe’ of a ‘30-million-word gap’ by the time children start school. EAL students also face challenges, typically having smaller vocabulary sizes than monolingual peers, which can limit reading comprehension and knowledge expression (Bialystok et al., 2010; Sun & Yin, 2020). Frequent reading may potentially close such ‘gaps’ (Krashen, 2004), increasing the quantity (e.g., number of words) and the quality (e.g., which words are used) of children’s language exposure (Rowe, 2012). For instance, Hayes and Ahrens (1988) found that children’s books contain 30.9 (SD 5.4) rare words per 1000 tokens, about three times that of adult-to-child conversations (*M* = 9.9). Meta-analyses have indicated that shared book reading can explain about 8% of language and reading performance in children under age 8 (Bus et al., 1995), and the amount of leisure reading can predict about 12% of preschoolers’ individual differences in oral skills (Mol & Bus, 2011).

During shared book reading, Evans et al., (2008) found that 3- to 5-year-old children spent most of the time looking at illustrations but that attention to orthography increased with age. Print-looking time occupied up to 25% of a child’s attentional focus during shared book reading, with children’s attention to the printed vocabulary enhanced by pointing. This print vocabulary that children are exposed to in picture books likely plays an important role in learning to read and language acquisition. However, we know little about the lexical properties of the words in this input. Previous research does not inform us which print words occur in picture books, limiting our understanding of early print exposure as well as the theoretical and computational modelling thereof. In the absence of lexical norms derived from a child’s print environment, norms from general (adult) corpora have been used, but as noted by Lété et al. (2004) this practice is problematic since such norms represent a different print environment.

The absence of lexical norms derived from children’s print for English is not the case for other languages, with several large-scale studies published in the current journal (e.g., Corral et al., 2009; Li et al., 2022; Schroeder et al., 2015). Their usefulness supports the need for such a database in English. Recent work in English has been underpowered due to the use of small corpora, ranging from 100 (Montag et al., 2015) to 160 picture books (Dawson et al., 2021). Although larger English datasets have been released, they are often difficult to access and are not updated regularly (Masterson et al., 2010; Wild et al., 2013). In the following paper, we begin with an overview of previous databases, in order to establish their design principles and importance in behavioural research. The review shows the limitations of existing lexical norms for experimental, educational, and computational modelling research with English-speaking children, motivating the current study. We then move on to describing an innovative method that extends the approach used to build subtlex-uk (van Heuven et al., 2014) through data mining speech-to-text algorithms. This results in a lexical database, the Children’s Picture Books Lexicon (cpb-lex), derived from a proxy for a sample of children’s picture books, many times larger than previous research. The method is reproducible and produces valid data. Validity is evaluated by correlating the new norms against previous databases as well as demonstrating the relationship between the new norms and age of acquisition (AoA) in regression models. The paper concludes with a demonstration of the utility of cpb-lex for extending modelling research on children’s print exposure and deficit input gaps. cpb-lex is freely available in the supplementary material.

## Currently available lexical databases of children’s books in English

This section reviews previous databases of lexical norms and demonstrates the need for updated, accessible norms in English. It also discusses their design features, methodologies and validation procedures. Arguably, the lexical statistics that currently constitute best practice in experimental psychology for adult input are the Subtlex norms, which for English are Subtlex-us (Brysbaert & New, 2009) and Subtlex-uk (Van Heuven et al., 2014). These are discussed here because they motivate methodological and design principles underpinning cpb-lex. Brysbaert and New (2009) derived Subtlex-us from 51 million words in 8388 subtitles of US television and movies. Data were crawled from a web repository, which had captured data via optical character recognition of DVD tracks. To validate the norms, variance was modelled with visual word recognition reaction times from the English Lexicon Project (Balota et al., 2007) and compared to alternative corpora. These norms explain more variance in lexical decision times than frequency norms based on older corpora (e.g. Kucera & Francis, 1967).

Subtlex-uk (van Heuven et al., 2014) was designed to better approximate the input of UK residents. Subtitles/closed captions from seven BBC channels were crawled, from which lexical statistics were computed. The researchers also collected data from two children’s channels, cbeebies and cbbc. The database contains frequencies for each type, lemma, and part of speech, as well as a contextual diversity measure (N programmes in which a particular word occurred). To guide selection of stimuli, for example in word frequency effect research, Subtlex-uk introduced the Zipf scale computed as:

$$\textrm{Zipf}=\log 10\left(\textrm{word}\ \textrm{frequency}\ \textrm{per}\ \textrm{million}\ \textrm{tokens}\right)+3$$

The Zipf is a log-transformed normed frequency scale that adds the integer 3 to range from 1 to 7. If a given word does not occur in a document, i.e., a raw frequency of zero, a Laplace transformation can inflate all words +1 per million and provide a hypothetical Zipf value. This process is valuable in contexts where researchers have reason to believe that in the overall pool of words from which a corpus is constructed, a word occurs but just has not been captured in the corpus sample. Subtlex-uk also contains bigram (two-word co-occurrence) frequencies. To validate Subtlex-uk, Zipf values for words were shown to be better predictors of lexical decision times by British participants than Subtlex-us, measured against British Lexicon Project reaction times (RTs; Keuleers et al., 2012). Of particular importance to the current study is the cbeebies and cbbc norms within Subtlex-uk. The cbeebies norms were derived from 5,848,083 words and contain 27,236 types, while the cbbc norms were derived from 13,612,278 words and contains 58,691 types. To support the validity of cbeebies and cbbc as representative of language input to children, frequency norms were shown to correlate strongly with those in the Children’s Printed Word Database (cpwd) (Stuart et al., 2003) and more strongly than with the overall Subtlex-uk frequencies. However, while cbeebies and cbbc are valuable datasets, they were not designed to represent the print environment of children or input from picture books.

The cpwd (Masterson et al., 2010; Stuart et al., 2003) was designed to capture print-based lexical statistics and contains 12,193 word types from 1011 books and approximately 995,927 words. The database existed online for researchers (https://www1.essex.ac.uk/psychology/cpwd/) but is no longer fully functional. The cpwd was derived from texts recommended for 5–9-year-old children in the UK. The sample frame was guided by a survey of schools in 1989 and includes readers used in schools (sample titles include *Breakthrough to Literacy, Oxford Reading Tree*, etc). Stuart et al. (2003) found that the cpwd was significantly different from adult corpora, for example, containing extensive orthographic representations of non-words, such as ‘whoosh’, variants for interjections: aaaaaah, aaaah, aaarrggh. Further, frequently encountered words were often not transparent grapheme-phoneme correspondences (e.g., one, was, put), which is surprising given that the texts were designed for early literacy. For example, the most frequent syllable structure in monosyllabic words within the most frequent 100 words was CVC (consonant-vowel-consonant) but only 41% had three letters. Stuart et al. (2003) concluded that this indicates the need for digraph instruction to assist students in decoding irregular forms. The researchers also considered indexes of gender such as pronoun frequency, reporting significant bias: 7711 male terms but only 3715 female. We explore this finding in relation to cpb-lex in later sections.

The largest existing English database is the Oxford Children’s Corpus (occ) (Wild et al., 2013). This is a so-called monitor corpus and is being updated regularly by adding books published by Oxford University Press (OUP). Access to the data is restricted, and it has been used mostly for commercial purposes, such as learner dictionaries. Researchers can apply to access the data, with projects vetted by OUP. Upon release, the occ contained approximately 30 million words from texts with recommended ages of 5–14. As far as the authors are aware, no frequency norms have ever been published, possibly for commercial reasons. The corpus includes fiction, comics, non-fiction and textbooks. It contains a supplementary sample of children’s writing (1.4 million words). Some sampling is from the public domain and dated, for example approximately 4.5 million words pre-1900, with texts such as *Pride and Prejudice*. The occ may thus be of limited use as a proxy for the print exposure of picture books for experimental or computational research.

## Lexical databases of children’s books in languages other than English

While lexical norms of early print exposure in English are limited, several have been developed for other languages. These cross-linguistic databases have a typical set of norms and features (e.g. type frequency, dispersion, contextual diversity, part of speech, length), which we also provide for cpb-lex. Escolex (Soares et al., 2014) was extracted from (European) Portuguese elementary and middle school (ages 6–11) textbooks constituting a 3.2-million-word corpus. It contains 48,381 types, which can be adjusted for developmental stages, since the 171 books sampled came from curricula with grade recommendations. The database reports grade frequency, overall word frequency, dispersion, frequency per million words, contextual diversity and a standard frequency index (SFI; computed by weighting log frequency against dispersion across texts). Other metrics include the number of letters, syllables, part(s) of speech, syllable structure and comparable frequencies in adult corpora. A French database, manulex (Lété et al., 2004), was derived from 1.9 million words of elementary school readers. manulex contains 48,886 types and provides frequencies, dispersion, SFI and adjusted frequencies for grade level, lemmas, part of speech and character length. The German-language childlex (Schroeder et al., 2015) was derived from 500 books and approximately 10 million words. The database reports word frequencies, dispersion, lemma, part of speech and neighbourhood size information. The Greek lexicon, Helexkids (Terzopoulos et al., 2017) is a graded database (grades 1–6) derived from 116 textbooks. It contains for 68,692 types, their Zipf values, frequency per million, lemma frequency, SFI, dispersion, contextual diversity and average orthographic Levenshtein distance. The Chinese Children’s Lexicon of Written Words (ccloww) (Li et al., 2022) contains norms for Chinese words and characters computed from 22.5 million words in 2131 children’s books. The sample includes fiction and academic texts sampled from school reading lists. The sampling frame contained grade level recommendations, allowing ccloww to provide overall frequencies and grade-level norms. The database reports contextual diversity, part of speech, lemma and word length information. Li et al. (2022) validate the norms by reporting strong correlations between Zipf values and adult lexical decision times. Regression models indicated that ccloww frequencies explained 12.11% more variance in lexical decision times in addition to Subtlex-ch frequencies (Cai & Brysbaert, 2010), interpreted as a processing speed advantage for words with early exposure.

## Pedagogically oriented frequency lists

Several frequency-based wordlists have been developed within educational research. While the primary purpose of such lists has been pedagogical, they have often been used in experimental work. An example is *The American Heritage Word Frequency Book* (Carroll et al., 1971) and *The Educator's Word Frequency Guide* (ewfg) (Zeno et al., 1995). In relation to the former, Carrol (1972) stated that “colleagues in psychology may find it of help in selecting words for use in experiments on verbal learning… However, the book was thought of primarily as a tool for teachers” (p. 11). For decades, two lists have been widely used in early literacy instruction, namely the Dolch and Fry 100 ‘sight word’ lists. These are intended to represent the most frequent words children are exposed to in print. Farrell et al. (2013) claim that in the US, most early literacy teachers use the Dolch or Fry high-frequency words in beginning reading instruction. These words have been incorporated into test batteries such as TOWRE-2 (Torgeson et al., 1999). The pedagogical argument has been that the most frequent 100 words in a child’s print exposure make up about half of all print words they encounter when learning to read, so the ability to recognise them automatically without decoding (i.e. by sight) is beneficial. Castles et al. (2018) conclude that in combination with phonics, learning a set of high-frequency words during emergent literacy may be valuable to reading fluency. Because the Dolch list was published in 1936 and the Fry list in 1957 (though updated with Carrol’s (Carroll, 1971) norms in 1980), the cpb-lex corpus allows for the creation of new lists that may be useful in combination with phonics.

## Modelling the lexical input from children’s picture books

Schroeder et al. (2015) note that developing lexical databases representing children’s reading input is essential for modelling their language environment. Recent examples of modelling studies that could be extended by a new resource include Montag et al., (2018) and Dawson et al., (2021), which used corpora of 100 and 160 picture books, respectively. These studies investigated the properties of early print and modelled the cumulative vocabulary exposure children might receive over time. They compared book vocabulary to child-directed speech (CDS) and modelled the additional lexical diversity contributed by picture books. It has been argued that lexical diversity is an important predictor in language development (Montag et al., 2018) and, to facilitate comparison with the previous studies (Dawson et al., 2021; Montag et al., 2018), the present study operationalises this variable as the type-to-token ratio; we do, however, acknowledge, that other measures may be more comprehensive (Yang et al., 2022).

Dawson et al. (2021) built a 160-book corpus and compared the data to adult utterances from childes, a repository of child-directed speech and child utterances (MacWhinney, 2001). The picture book corpus contains 319,435 words, with sampling guided by impressions of popularity in online webpages (e.g., bestseller lists). It is available at https://osf.io/zta29/, with the words of anonymised texts randomised (i.e., a bag of words), given intellectual property restrictions. Dawson et al. (2021) model the cumulative number of new types encountered over time in CDS and picture books and report that, given equal input (in tokens), children would encounter many more new types from books than CDS. Comparing word length, part-of-speech distributions and morphological complexity, picture books were found to have significantly more content words, more nouns and adjectives, but fewer verbs, indicating denser information content than in speech. The 1000 most frequent word types in subtlex-uk (including function words) overlapped more with CDS (79%) than picture books (72%). Book keywords (i.e., types that were significantly more frequent in books as compared to CDS according to the results of a log-likelihood test) were found to have higher average ages of acquisition, arousal and positive sentiment values, but lower concreteness. Dawson et al. (2021) conclude that “books provide more concentrated access to the types of words that are not supported by direct sensory experience, along with the linguistic and contextual information needed to support learning and consolidation” (p. 25).

Montag et al. (2015, 2018) explored the relationship between type input, token input and learning environments using a 100-book corpus (68,103 words), with comparisons to 4432 transcripts of conversations (6.5 million words) from childes. Montag et al. (2018) simulated multiple learning environments with type deficits in the CDS. Children may receive less lexically diverse input from CDS due to limited token input (Hart & Risley, 2003), conceptions about how best to talk to children (i.e. reduced complexity), or variability in caregivers’ vocabulary sizes. From the CDS proxy, a year-long lexical diversity curve was created that plotted cumulative new types as a function of token input (using approximately 5 million words as the token input parameter). This baseline was penalised to create two impoverished curves with 10% and 20% of the types randomly removed from the underlying corpus. To model the impact of books, Montag et al. (2018) added to the CDS corpus the types in the 100 books and regenerated the curves. Results indicated that lexical diversity over the year increased, reducing the 20% deficit curve by 6% and the 10% deficit curve by 4%. The researchers concluded that input from books would have greater benefits for children with more impoverished input. They argued that adding a small number of books to the input a child receives could potentially contribute to closing deficit gaps. Given the small corpus, it was not possible to model how many books might close how much of a gap, but Montag et al. (2018) explores the question by adding the 100 books to data pools of different sizes representing different time spans, i.e., 650,000 words of CDS representing a month of input, with 10, 50, 100 books added. The 100 books in a month model, approximately three books a day, increased type exposure by 16.5%, which Montag et al. (2018) suggests may be realistic for some children.

The most widely known figures for vocabulary input deficit gaps are Hart and Risley’s (Hart & Risley, 2003) ‘30-million-word gap’ amongst different socio-economic classes (professional, working class and welfare). In 1318 recorded conversations, welfare children heard on average 616 words per hour, working class 1251 and professional 2153. Simulating a 14-hour day, children from professional families received 11 million words of input per year; working class 6.5 million and welfare 3.2 million. By school entry, the modelling suggested approximately 30 million fewer words of input to the welfare children compared to the professional class. Extensive reading has been one avenue of research into how we might ‘close the gap’ (Krashen, 2004). Logan et al. (2019), for example, computed from 60 picture books that reading one book a day could contribute an additional 77,896 word tokens per year, and five books per day could potentially add an additional 1.4 million words by the time children start school. The following study will demonstrate how cpb-lex can build upon these modelling studies.

## The current study

This study releases the Children’s Picture Books Lexicon (cpb-lex), derived from a proxy for 2146 picture books and constituting the largest lexical database of early print exposure yet developed. Cpb-lex is released as three spreadsheets: the cpb-lex (lexicon) contains ranked wordforms, frequency norms, Zipf values, lemma, part- of speech (POS), two measures of dispersion (Coefficient of Variation and Deviation of Proportions) and a contextual diversity measure (*N* texts) for each type. For each wordform, reported are its number of letters, phonemes, age of acquisition and frequencies in comparable databases such as cbbc, cbeebies and Dawson et al.’s (2021) picture book corpus. Text-level statistics required for modelling studies such as those mentioned in the previous section are provided in another spreadsheet, the cpb-lex (document-matrix). This contains for 2146 anonymised texts their number of words (tokens), types, mean word length (in characters) and lexical diversity measured by two normalised type–token ratios: a standardised type–token ratio (STRR: unique types per 100 tokens) and a moving average ratio (MATTR: unique types averaged across a moving window of 100 tokens). Tokens are represented in an abstract document-term matrix for other researchers. A third spreadsheet, cpb-lex (bigrams), contains a bigram lexicon in the form of a frequency ranked list enriched with forward and backward transitional probabilities, information theoretic surprisal values, Zipf values for the bigram and POS information. Cpb-lex provides data for a wide range of experimental, computational and educational research. After describing the methodology and development of cpb-lex, the paper demonstrates several contributions it makes to the research programmes reviewed above.

## Method

Cpb-lex was built through the incorporation of a Google API (application programming interface) to access YouTube videos with additional Creative Commons APIs to extract closed captions/subtitle transcripts associated with videos of picture books being read aloud and shared publicly on social media. Lexical statistics were extracted from the transcripts. All code is open and in the public OSF repository: https://tinyurl.com/yc8dnsfm. The method leverages the new automatic speech-to-text function of YouTube. In recent years, automatic speech recognition (ASR) technology has been added to YouTube which automatically provides a transcription of speech content in closed captions (CC), as well as possible manually uploaded subtitles if applicable and available. The ASR technology is robust in accuracy, with recent work reporting 98% accuracy (Millett, 2021), though it is negatively affected by mispronunciations, background noise, dialects and accents. This pool of ASR captions formed the underlying data from which lexical information was extracted for cpb-lex. Also crawled were metadata such as video length and book title (if available). Python code searched YouTube channels and links identified through search terms ‘picture books, read aloud’. Channels with picture book readings as their primary focus were used as input to the code, which automatically retrieved a list of videos on each channel and accessed any captions. Retrieval of video links was based on Google/YouTube developer API code available here: https://www.googleapis.com/youtube. The code to list videos is here: https://developers.google.com/youtube/v3/docs/channels/list, and to request captions, here: https://developers.google.com/youtube/v3/docs/captions/download. Further code from a Creative Commons API was adapted to extract transcripts (https://github.com/jdepoix/youtube-transcript-api.) Captions were saved to a .txt file for pre-processing and to generate lexical statistics, then destroyed. Following Subtlex and consistent with internationally recognised fair dealing provisions for educational research, cpb-lex does not store, reproduce or redistribute the textual content of any data sources. It consists of the statistics of words and bigrams computed from public transmissions (Van Heuven et al., 2014, p. 1178), and lexical content for research is abstractly represented within a document-term matrix object.

Pre-processing and cleaning were undertaken to reduce noise. ASR metalanguage such as ‘[music]’ and ‘[applause]’ was deleted using string-searching algorithms (regular expressions/regex). Non-book readings and duplicates were removed though manual inspection. Transcripts usually had a welcome and goodbye statement, with book reading between. Book text was isolated by manually deleting unwanted material at the start and end of transcripts. Three research assistants were trained to complete the task. To undertake calibration, each received a sample of 20 transcripts and independently identified the start and end of book text, followed by a meeting with the research team to reach consensus before cleaning remaining data. All minor conflicts were resolved during the team discussion. Inspection of the transcripts did not suggest that readers made additional comments during readings and rather read from beginning to end of the story.

To develop the cpb-lex (lexicon), a frequency list of wordforms (tokens) was generated through lancsbox (Brezina, & Platt, 2023, available here: https://lancsbox.lancs.ac.uk/). This tool was chosen due to being an advanced corpus handling tool which could improve workflow efficiency by providing automatic computation of multiple metrics of interest to this study. It is open-access to the research community, which improves the reproducibility of the current research and allows easy user-defined token definition. We defined tokens to include hyphenated words and contracted forms, e.g., *it’s*. In post-processing, we found approximately 350 low-frequency types with character encoding flaws, causing them to be counted separately from their matching word (e.g. wow,┬á), so their frequencies were manually added to the correct type. Zipf values were computed using the formula in Van Heuven et al. (2014), noted above. Values were not Laplace-transformed by +1, to avoid assuming that unattested forms were part of children’s vocabulary exposure from picture books. Cpb-lex (lexicon) reports two dispersion measures, the coefficient of variation (CoV) and deviation of proportions (DP), recommended as best practice and found to correlate better than alternatives with lexical decision times (Brezina, 2018; Gries, 2019). Researchers are able to compute any preferred dispersion measures from the raw statistics provided in cpb-lex. The CoV is computed as:

$$\textrm{CoV}=\textrm{SD}/\textrm{M}\ \textrm{and}\ \max =\surd \textrm{n}\left(\textrm{corpus}\ \textrm{documents}\hbox{--} 1\right)$$

That is, the CoV is the standard deviation (SD) divided by the mean (M) of a token frequency across corpus texts, and the maximum value is the square root of *N* texts minus 1. DP is scaled 0–1, with values closer to zero indicating a more even distribution in the corpus. It is computed as follows:

$$\textrm{DP}=\Sigma\ \textrm{ABS}\left(\textrm{O}\hbox{--} \textrm{E}\right)/2$$

DP is the sum of the absolute difference between observed (O) and expected distributions (E) divided by 2. The expected value is obtained by dividing the tokens in each text by the overall tokens in the corpus.

To develop the cpb-lex (document-matrix), multiple text-level metrics were computed in lancsbox. The tool provided for each transcript statistical information for the number of types and tokens. Because type–token ratios are influenced by text length, two normed values were computed: a standardised type–token ratio (STTR) which divided each text into 100 token parts and computed an average ratio across the parts, and a moving average type–token ratio (MATTR) which computed an average for each text from a moving window of 100 tokens (Brezina, & Platt, 2023). Statistics were aligned with timestamp information (length of video in minutes). Data were converted to a document-term matrix for the cpb-lex (document-matrix). dtms are abstract mathematical representations of documents and terms (i.e. types and their token frequency), widely used in natural language processing (NLP). Lemmatisation and POS tagging was performed in Python with Spacy (https://spacy.io/). Spacy is currently one of the most accurate POS tagging systems, reporting 97.40% accuracy (https://spacy.io/usage/facts-figures). Commenting on the challenges of POS tagging in Subtlex, Brysbaert et al. (2012) suggest that machine-generated tags constitute useful information, despite errors, and while ideally 100% accuracy is desired, manual correction is ruled out by the prohibitive costs involved.

To develop cpb-lex (bigrams), bigrams were identified and their frequencies computed in lancsbox. Based on these raw frequencies, forward and backward transitional probability were computed in Excel for each bigram (word A, word B) using the formulae:

$$\textrm{Fwd}\ \textrm{TP}\ \left(\textrm{A}|\textrm{B}\right)=\textrm{AB}/\textrm{A};\textrm{Bwd}\kern0.5em \textrm{TP}\ \left(\textrm{B}|\textrm{A}\right)=\textrm{AB}/\textrm{B}$$

That is, forward TP is the probability of word B occurring after word A as a function of the overall frequency of the bigram divided by how many times word A occurs with other words. The formulae are reversed for backward TP. Also computed were surprisal values (Levy, 2013), an information theoretic measure of the amount of information in word B given word A, computed as:

$$\textrm{surprisal}\left(\textrm{B}\right)=-\log 2\left(\textrm{FwdTP}\right)$$

This surprisal formula has been found to be a strong predictor of reading times cross-linguistically (De Varda & Marelli, 2022). To assist researchers in isolating specific types of bigrams, such as content word combinations, a supplementary bigram lexicon was generated from the POS data. For this, transitional probability and surprisal values were computed from the POS single-word lists found in the cpb-lex (lexicon). Cpb-lex (bigrams) provides raw bigram counts, from which other associative measures (e.g., mutual information) can be computed.

To validate cpb-lex, several analyses were planned, which included the computing of correlations between the new frequency norms and those in prior resources, as was done with cbeebies and cbbc to indicate overlap with the cpwd. We hypothesised that cpb-lex would be a good predictor in regression models of the variance in AoA, and further, that if it is an improvement to alternatives, it would perform better against AoA than existing child input lexical norms.

## Results and discussion

The above methods produced cpb-lex from approximately 1,120,778 tokens across 2146 unique picture book readings. The lexicon contains approximately 25,585 types. Cpb-lex is provided in full at the OSF repository (https://tinyurl.com/4este73c). This section explores the validity of cpb-lex and demonstrates how it can extend the research reviewed in previous sections. First, we note that the pool from which the lexical statistics were derived is many times larger than recent comparable picture book datasets, such as Montag et al. (2018) and Dawson et al. (2021), yet the statistical information draws from similar books. A sample of titles underlying the lexical statistics is provided in Table 1, and this sample suggests that cpb-lex is representative in terms of common input for emergent readers. For researchers to have a clear idea of data underlying cpb-lex, hyperlinks to source channels are provided in the OSF supplementary materials.

**Table 1:** Example titles underlying lexical statistics

*Go To Sleep, Groundhog* *Scaredy Squirrel* *Monster Knows Manners* *The Bunny Who Found Easter* *Good-Night, Owl!* *Hey, That's MY Monster!*	*The Day the Crayons Came Home* *The Gingerbread Man* *The Three Billy Goats Gruff* *Llama Llama Holiday Drama* *Thomas and the Big, Big Bridge* *Lola Goes to School*

### Properties of the cpb-lex (lexicon)

**Fig. 1:**
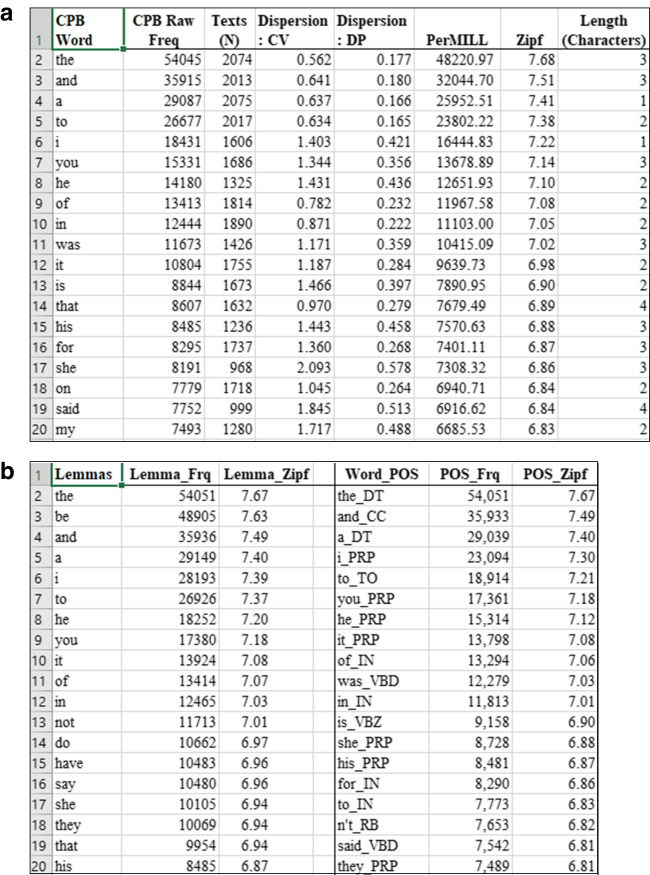
**a** The cpb-lex (20 most frequent wordforms) **b** The cpb-lex (20 most frequent lemmas and parts of speech)

Figure 1(a and b) show the organisation and information in the cpb-lex (lexicon), the central database of lexical statistics.

Figure 1a shows the cpb-lex (lexicon) is organised by rows for each wordform ranked by frequency. Columns are given for raw frequency, the number of texts in which the word occurs (a measure of contextual diversity), dispersion values, frequency per million words and frequency normed on the Zipf scale. The number of wordforms is 25,585 types, a result of the large sample (Van Heuven et al., 2014), which allows cpb-lex to provide statistical information for almost twice as many types as Dawson et al. (2021) and the cpwd (Stuart et al., 2003) (both approx. 13,000). The number of types is close to cbeebies’ approximately 27,236. Figure 1b shows ranked frequency of lemmas and wordforms tagged for part of speech. These are in a separate tab within the spreadsheet. Sorting the word_pos column alphabetically groups related words and their frequencies together, for example wordforms that may function as both nouns and verbs depending on use. Future researchers may benefit from quick access to values for words in this corpus shared in common with previous research databases, so the lexicon contains columns aligned with the wordforms as follows: Zipf values from cbeebies, cbbc, subtlex-uk (as reported in van Heuven et al., 2014), Dawson et al’s (2021) picture book corpus (dpb); several age-of-acquisition (AoA) values: AoA (Kup) (as reported in Kuperman et al., 2012); AoA (test-based), AoA (rating) and AoA (LWV) (as reported in Brysbaert & Biemiller, 2017); word prevalence, character length (NLetters), number of phonemes (NPhon), morphemes (NMorph), syllables (NSyll) (as reported in Brysbaert et al., 2019), LDT_RT (reaction times, lexical decision tasks) and NMG_RT word naming times (as reported in Balota et al., 2007); phonological/orthographic family size (Ortho_N, Phono_N); and average phonological/orthographic Levenshtein distances (OLD, PLD) (Brysbaert et al., 2019) . Details on these widely used norms can be found in associated references. Table 2 summarises mean values for wordforms in the cpb-lex for frequency, length, age of acquisition and processing.

**Table 2:** Summary statistics for the cpb-lex (lexicon)

*Frequency*	*Length*	*Age of acquisition*	*Processing*	*Dispersion*
Zipf	NSyll	AoA (test-based)	LDT_RT	N Texts
3.56 (.68)	2.00 (.89)	5.55 (3.50)	687 (82)	18 (89.23)
Per/mil	NMorph	AoA (LWV)	NMG_RT	Coeff. variation (CV)
39.85 (521.99)	1.82 (.71)	6.20 (3.10)	652 (61)	33.23 (13.62)
Raw	NPhon	AoA (Kup)	Prevalence	Dev. proportions (DP)
43.81 (585.04)	5.59 (1.88)	8.29 (2.53)	2.26 (.24)	.998 (.047)
	NLetters	AoA (Rating)		
	6.84 (2.28)	7.31 (3.26)		

*Standard deviation in brackets

Table 2 indicates words in cpb-lex are relatively well dispersed and occur across a number of books. The data on length suggest they are generally shorter and less complex than adult norms, cf. subtlex-uk: Nsyll 2.4 vs 2.00; Nphon 6.5 vs 5.59; Nmorph 2.1 vs 1.82; NLetters 7.84 vs 6.84. Prevalence and processing times indicate they are more widely known and processed faster by adult speakers compared to the overall set of words Brysbaert et al. (2019): i.e., overall prevalence: 2.15 vs 2.26 cpb-lex subset; LDT_RT 740 ms vs 687 ms; NMG 688 ms vs 652 ms). Compared to the 44,000 words in Brysbaert and Biemiller (2017), cpb-lex words have an earlier average age of acquisition, AoA (test-based) 8.66 vs 5.55, AoA (rating), 9.5 vs 7.31, AoA (LWV) 9.08 vs 6.20; and similarly, the 31,000 Kuperman et al. (2012) norms, AoA (Kup) 11.00, vs 8.29. From these numerical differences, it seems feasible that cpb-lex does indeed reflect early print environments, i.e., earlier AoA, shorter and simpler than the general pool of words in the English language.

Validity evidence for previous databases has come from correlations with existing norms; for example, cbeebies was correlated with cpwd (Van Heuven et al., 2014) to show it captured a similar construct. Other evidence for validity has come from demonstrating that new norms explain more variance than existing alternatives on a criterion, for example on RT norms (Brysbaert & New, 2009). If cpb-lex reflects early print exposure, it should be a good predictor of AoA norms. Further, cpb-lex should perform better against AoA than existing child input lexical norms, if it is an improvement to alternatives. We explored the relationship between cpb-lex and multiple AoA norms: AoA (Kup), which are age of acquisition estimates from adult participants (Kuperman et al., 2012); AoA (Rating), a version of the Kuperman et al. (2012) norms with additional words added from other databases and aligned through linear regressions and adjusted for frequency; AoA (LWV), which are estimates derived from direct testing of children in US schools prior to 1980; and AoA (Test-based), an updated version of the LWV with values adjusted by some recent age-of-acquisition research findings on subsets of the target words. The AoA (Rating), AoA (LWV) and AoA (Test-based) values were taken from Brysbaert and Biemiller (2017). Table 3 reports Spearman correlations between four AoA norms and wordform Zipf values from cpb-lex, with comparisons to cbeebies, cbbc, dpb (the Picture Book Corpus by Dawson et al., 2021) and subtlex-uk. Correlations were computed on a listwise deletion basis and excluded Laplace-transformed Zipf values for non-occurring targets in cbeebies and cbbc, and the RTs were log-transformed. All correlations in Table 3 are significant at *p* < 0.001 (with Bonferroni correction).

**Table 3:** Correlations of word frequency norms with age of acquisition, RT and prevalence

	*cpb-lex*	subtlex-uk	cbeebies	cbbc	dpb
subtlex-uk	0.684				
cbeebies	0.801	0.655			
cbbc	0.751	0.908	0.743		
dpb	0.776	0.621	0.734	0.679	
AoA (Kup)	−0.670	−0.402	−0.625	−0.499	−0.586
AoA (Rating)	−0.685	−0.405	−0.656	−0.507	−0.611
AoA (Test-based)	−0.519	−0.362	−0.480	−0.397	−0.449
AoA (LWV)	−0.420	−0.291	−0.389	−0.315	−0.363
Prevalence	0.216	0.327	0.186	0.298	0.173
LDT_ RT	−0.489	−0.541	−0.422	−0.538	−0.403
NMG_ RT	−0.351	−0.353	−0.309	−0.359	−0.316

*All correlations significant at *p* < 0.001

Noteworthy is that cpb-lex has strong correlations with previous child input norms, cbeebies, cbbc, dpb and a weaker correlation to subtlex-uk, suggesting that it measures a construct similar to previous children’s lexicons (van Heuven et al., 2014). Cpb-lex frequencies are more strongly correlated with AoA than cbeebies, Dawson et al.’s (2021) DPB, and general frequency in English as measured by subtlex-uk. The inverse correlations with AoA norms indicates that higher-frequency words in this input correlate with earlier ages of acquisition. These data taken together suggest that cpb-lex represents early print exposure and improves upon previous resources in capturing this language environment.

**Fig. 2:**
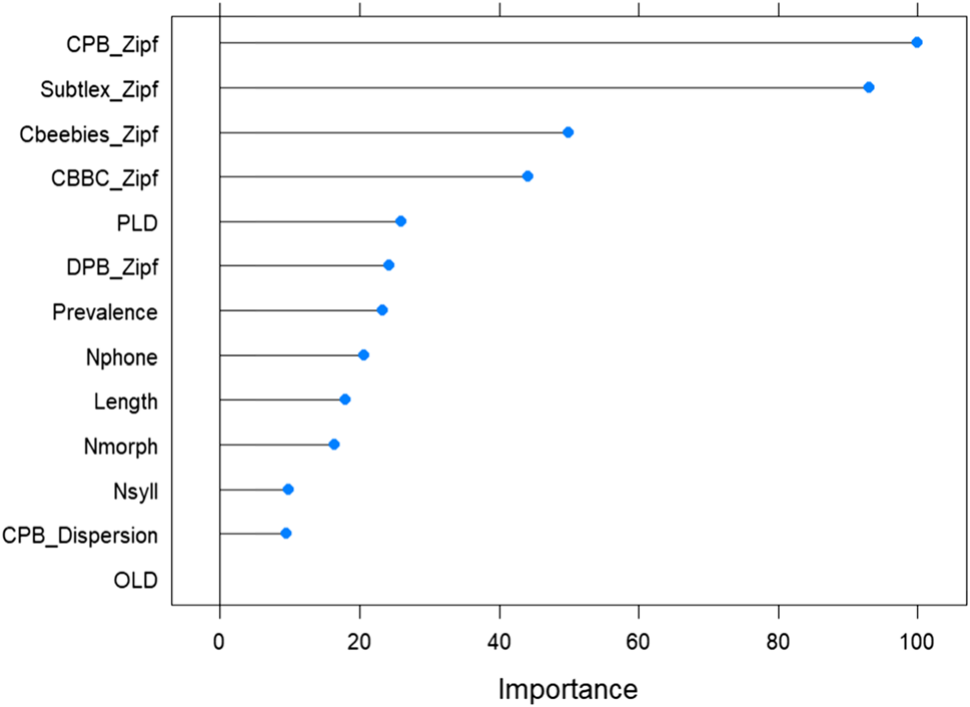
Regression model AoA, visualisation panel

Moreover, cpb-lex explains more variance in words’ age-of-acquisition ratings compared to the other word frequency metrics, and psycholinguistic features. Using an elastic net regression model (glmnet in R caret package with repeated cross-validation), all five word frequency measures (Zipf values from cpb-lex, cbeebies, cbbc, subtlex-uk, dpb), along with six psycholinguistic features commonly used as control variables (word length, number of phonemes, syllables and morphemes, and orthographic and phonological Levenshtein distance) and word prevalence, were entered together as predictors of AoA (Kuperman et al., 2012). The psycholinguistic predictors were scaled, and only words with values across all measures were included in the model. To examine how much each predictor contributed to the outcome, relative variable importance was estimated (derived from weighted sums of regression coefficients and error reduction related to the variable; Zou & Hastie, 2005), with values ranging between 0 and 100 (Kuhn, 2010) as presented in Fig. 2. As shown, Cpb-lex has higher importance than other variables, including the other child corpus measures of word frequency (β = −1.078, vs cbeebies = −0.497, cbbc = −0.430, dpb = −0.221; *R*^2^ = 0.479). In addition, while subtlex ranks second in variable importance, its coefficient is positive (0.919), in contrast to the other word frequency norms which all hold a negative relation to AoA. A negative relation is expected, because higher-frequency words (with a greater Zipf value) should have lower (earlier) AoA. Lower-frequency words having earlier AoA, on the other hand, seems counterintuitive, and could be explained by elementary words that are used often with child input but diminish for adults’ use (as reflected in the subtlex norms).

### Text-level information in the cpb-lex (document-matrix)

**Fig. 3:**
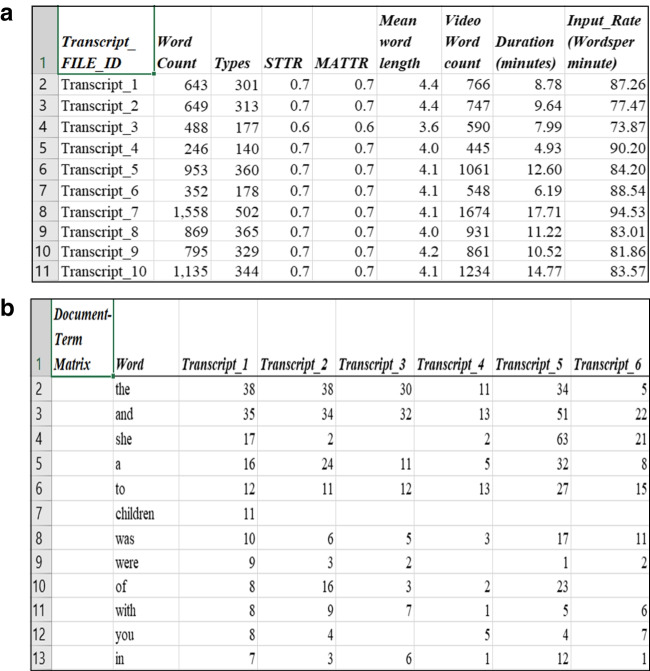
**a** The cpb-lex (document-matrix), document level statistics **b** The cpb-lex (document-matrix), DTM Object

The cpb-lex (document-matrix) is a document-term matrix (dtm) that contains statistical information at the text level. This information is essential for research such as the computational modelling in Montag et al. (2018) and Dawson et al. (2021) and has many other research applications. Figure 3a and b show the organisation of the Document-Matrix and the information it provides.

The matrix includes word count, unique types, average word length, STTR, MATTR, video duration and approximate input rate in words per minute. The dtm provides a word in each row, frequency in each cell and document association per column. Table 4 provides summary statistics for the cpb-lex (document-matrix).

**Table 4:** Summary statistics for the cpb-lex (document-matrix)

*N documents:* *2146*	*Length* *(tokens)*	*Types*	*STTR*	*MATTR*	*Time* *(min)*	*Input rate* (wpm*)
Mean	522.27	215.72	0.67	0.67	5.88	95.43
Standard dev.	411.11	13.52	0.08	0.08	3.28	28.94
Min.	21.00	10.00	0.21	0.20	0.80	9.60
Max.	4921.00	1222.00	0.95	0.96	32.79	252.24

*wpm: Words per minute

Table 4 indicates an average length of 522 tokens per book, with a wide range of word count and input rates. Average reading time is 5.8 minutes. As far as the authors are aware, this is the first reading rate estimate for children’s picture books based on a large sample. In 214 hours, all 2146 books could be read. One way to interpret this is in the context of previous research. Montag et al.’s (2018) modelling indicated a 16.5% increase in the lexical diversity of a child’s input with three books per day over a month, with speculation on whether the number would be realistically attainable. Given the new input rate estimation, this would be only about 18 minutes of reading a day. Of course, this is a simplified model, and not every book will be as rich as the next in its lexical diversity, but the data suggest that in general, an inordinate amount of time reading is not required to provide a child with improved exposure to rich vocabulary.

While there are few comparison points in the research record, the input rate appears to be faster than reported rates for children’s entertainment such as Sesame Street at 60 wpm (Spanos & Smith, 1990) and lower than adult speech rates in the typical range of 120–180 wpm (Brysbaert, 2019). This may support proposals that one benefit of shared book reading is that the input rate is typically higher than other input directed toward the child. Inspection of the corpus indicated 24 texts above 2000 words (approx. 1% of the corpus). We considered removing longer texts. For example, at this threshold, Logan et al. (2019) removed picture books from their corpus as outliers. However, we decided against this after inspecting them. The longest book (indicated in Table 4 as having 4921 tokens) was retrieved online and was a reading of *George’s Marvelous Medicine* by Roald Dahl. While it had more text on the page than many other books, it was read aloud in the same format and contained illustrations on each page. Other than being longer, we could think of no reason to exclude longer books. One reason for providing the dtm is so researchers can adapt the cpb-lex for their projects and can exclude longer texts if desired or investigate potentially interesting relationships such as between rate of input, length, AoA and other variables. The correlation between book length (word count) and input rate is *r* = .62, *p* < .001, the same for unique types and input rate *r* = 0.62, *p* < .001, indicating that as the texts get longer and more lexically diverse, reading rate increases.

### The cpb-lex (bigrams)

**Fig. 4:**
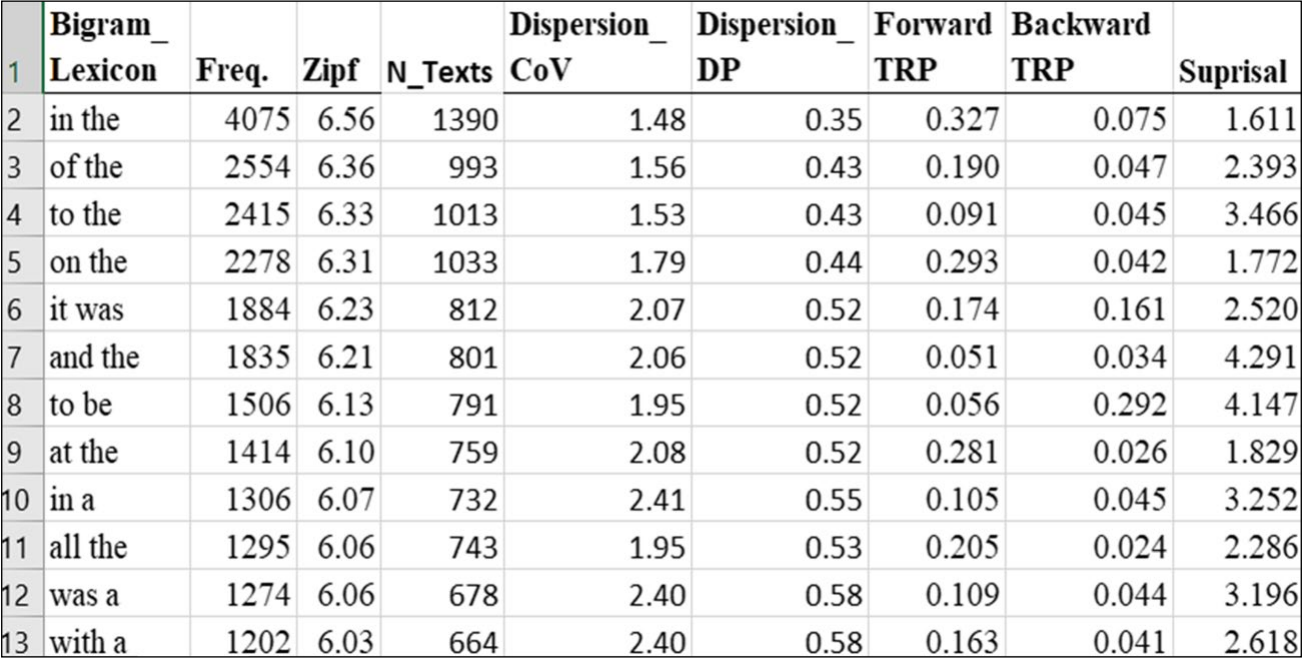
Organisation of the cpb-lex (bigrams)

An overview of the cpb-lex (bigrams) database is given in Fig. 4.

**Fig. 5:**
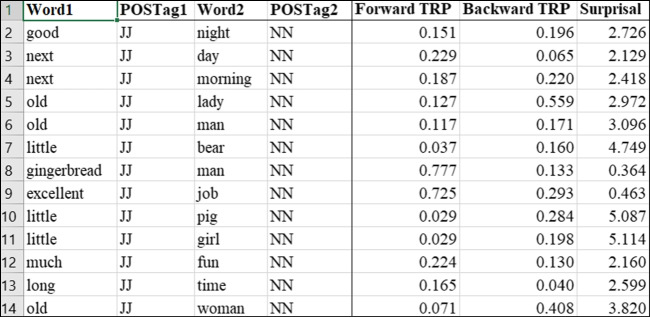
Organisation of the cpb-lex (bigrams, pos)

The dataset contains 351,292 bigrams. As far as the authors are aware, only subtlex-uk contains bigram norms; however, those data do not report information for cbeebies or cbbc. Cpb-lex (bigrams) therefore constitutes the only child print norms for bigrams. As shown in Fig. 3, bigrams are ranked by frequency, with one bigram per row, and columns for raw frequency, the number of text occurrences, dispersion and Zipf values. We also provide three association metrics: forward and backward transitional probabilities and surprisal. Figure 5 shows the part-of-speech supplement in a separate tab within cpb-lex (bigrams).

As depicted in Fig. 5, the database can be sorted to find particular targets, such as samples of JJ (adjective) and NN (noun) bigrams. With this information, researchers might select stimuli controlled along certain variables such as part of speech, while varying values such as surprisal or transitional probabilities. In Fig. 4, for example, the adjective-noun combination *little girl* has higher surprisal values for word 2 than the adjective-noun combination *excellent job*, because less information is provided by *little* to predict the next word in comparison to *excellent*; while *old lady* and *old woman* have higher backward transitional probability than *old man*, indicating *lady* is more often preceded by *old* than is *man*.

### Deriving high-frequency wordlists from cpb-lex

As reviewed earlier, the so-called sight words of Dolch and Fry are widely used representations of the most frequent 100 words children are exposed to when learning to read. They are used internationally in classrooms and experimental batteries (Torgeson et al., 1999). They were published decades ago, and cpb-lex offers a potentially better alternative. Table 5 presents a high-frequency word list derived from the 100 most frequent words of cpb-lex. These are rank-ordered by frequency in cpb-lex, with two additional columns with the word’s comparable ranking in the Fry and Dolch lists, while ‘-’ indicates absence. This comparison reveals substantial differences between these lists. This may indicate that the previously published lists are outdated, and an updated list is in order.

**Table 5:** 100 highest-frequency words in cpb-lex compared to Dolch and Fry

cpb-lex Rank	cpb-lex Word	Fry rank	Dolch rank	cpb-lex Rank	cpb-lex Word	Fry rank	Dolch rank	cpb-lex Rank	cpb-lex Word	Fry rank	Dolch rank
1	the	1	1	34	one	27	98	67	or	26	70
2	and	3	13	35	when	37	38	68	down	93	14
3	a	4	25	36	what	33	50	69	go	74	-
4	to	5	7	37	your	38	-	70	big	-	-
5	I	20	31	38	like	65	92	71	don't	-	75
6	you	8	37	39	have	24	-	72	by	29	-
7	he	11	19	40	not	32	68	73	how	48	81
8	of	2	49	41	out	55	-	74	back	-	-
9	in	6	55	42	little	-	8	75	into	67	15
10	was	12	61	43	do	47	20	76	about	54	57
11	it	10	43	44	then	57	2	77	would	63	-
12	is	7	-	45	can	39	26	78	some	61	-
13	that	9	79	46	had	28	-	79	who	87	100
14	his	18	73	47	there	41	-	80	know	-	87
15	for	13	91	48	no	77	-	81	good	-	45
16	she	46	85	49	just	-	21	82	an	43	-
17	on	14	97	50	day	94	-	83	look	70	-
18	said	40	67	51	now	90	170	84	make	64	16
19	my	81	-	52	it's	-	-	85	right	-	93
20	with	17	-	53	from	25	39	86	more	72	-
21	but	31	-	54	were	35	74	87	love	-	-
22	they	19	-	55	him	66	127	88	our	-	-
23	all	34	-	56	I'm	-	-	89	did	95	44
24	her	62	-	57	will	51	-	90	way	78	-
25	so	60	56	58	see	75	62	91	here	-	-
26	be	22	-	59	them	58	86	92	new	-	-
27	we	36	-	60	if	50	-	93	over	-	-
28	at	21	-	61	time	68	-	94	night	-	-
29	up	52	-	62	their	49	-	95	made	98	83
30	me	-	-	63	too	-	-	96	even	-	-
31	as	16	-	64	oh	-	-	97	come	97	-
32	are	15	-	65	get	96	80	98	says	-	-
33	this	23	-	66	could	79	32	99	come	97	-
								100	home	-	-

Table 5 shows that 23 of Fry’s 100 words and 22 of Dolch’s are absent from the cpb-lex’s 100 most frequent, and frequency order largely differs between the lists. This suggests that the older lists may no longer be fit for purpose as representations of the high-frequency print words children first encounter. Similarly, a comparison of the most frequent nouns in cpb-lex (tagged NN [noun, singular], NNS [noun, plural], NNP [proper noun, singular]) to Dolch’s frequent nouns list (see Appendix 1 in the OSF supplementary materials) found that only 37 words overlapped. We suggest that cpb-lex is a more adequate representation of the print environment during emergent literacy. Consistent with Dawson et al. (2021) and Wild et al. (2013), the high-frequency words of cpb-lex in Table 5 and the Appendix reflect positive sentiment, family and the natural world from the perspective of the child: *home, family, mother, father, parents, cat, bear, moon, sky, tree, gold, monster, bed.* As Stuart et al. (2003) found in the cpwd, differences exist in male and female terms: ‘he’ occurs 12,651 times per million words, ‘his’ 7570, ‘she’ 7308 and ‘her’ 5318. In Appendix 1, ‘mom’ and ‘mother’ are more frequent than ‘dad’ and ‘father’, and ‘boy’ is more frequent than ‘girl’; however, ‘man’ is one of the top 20 nouns in children’s picture books, yet ‘woman’ is not within the top 100. Stuart et al.’s (2003) conclusion based on texts for children 30–40 years ago remains the case: “male protagonists are still the order of the day” (p. 594).

### Extending modelling studies of lexical diversity through cpb-lex

This final section builds on the lexical diversity modelling of Montag et al. (2015, 2018) and Dawson et al. (2021) and is intended to show the added value of cpb-lex. These previous studies found greater lexical diversity in children’s picture books than child-directed speech and modelled additional type exposure through picture books and how much this additional input might make up for deficits in input. For example, Montag et al. (2018) modelled the effect on cumulative type exposure of adding 100 books to a cds-proxy of 5 million words extracted from childes (MacWhinney, 2001), set as a parameter to represent 1 year of input. The researchers note that because there are limited types in only 100 books, their models may underestimate any effect. Since cpb-lex is larger, the research can be extended. Models were built for hypothetical children using the parameters of Hart and Risley’s (2003) ‘30-million-word gap’ figures, i.e. welfare children: 3.2 million words of CDS input per year, working class 6.3 million, professional 11 million. Following the previous work, a cds-proxy was created by extracting adult utterances from 6353 transcripts (approx. 11 million words) in the UK/US directories of childes (MacWhinney, 2001) (individual corpora are cited in supplementary materials). In childes, transcripts capture single speech events such as conversations. Mean tokens of the cds-proxy transcripts were 1737 words; therefore, the daily Hart and Risley (2003) figures were approximated as follows: welfare: 8767 words per day ≈ 5 transcripts; working class: 17,514 per day ≈ 10 transcripts; professional: 30,142 per day ≈ 17 transcripts. Indicated in Table 6, these parameters provided a close approximation of Hart and Risley’s (2003) year-long input.

**Table 6:** Type exposure at 365 days (cumulative)

Types	CDS Types	+ 1 book	+ 2 books	+ 3 books	+ 4 books	+ 5 books
Welfare	24,523	27,966	30,649	32,821	34,620	36,424
Working	33,671	36,883	39,038	41,140	42,803	44,308
Professional	44,086	46,567	48,495	50,292	51,822	53,211
Tokens	CDS Tokens	+ 1 book	+ 2 books	+ 3 books	+ 4 books	+ 5 books
Welfare	3,201,884	3,370,662	3,549,798	3,746,155	3,901,688	4,130,136
Working	6,333,962	6,532,077	6,689,588	6,910,647	7,118,577	7,305,342
Professional	10,779,153	10,969,147	11,156,321	11,348,563	11,549,079	11,734,778

A year-long model of cumulative type exposure was developed, following Montag et al. (2018). Python code was written to model exposure from the cds-proxy and compute the number of new types each of the three groups would encounter each day up to 365, creating for each group a cumulative exposure curve (code available here: https://tinyurl.com/4twspx3t). For each simulation, a hypothetical child was exposed to *n* random transcripts from the cds-proxy per day, where *n* was chosen based on the group being modelled. To these input curves, types from a random sample of 1–5 ‘books’ per day were added to see the effect on lexical diversity (Logan et al., 2019). Unlike previous studies, which took random samples of 100 words from books, given the larger size of cpb-lex, we were able to sample whole books, which arguably improves ecological validity. The code averaged the type exposure from 30 simulations, and this was taken as the data point each day in the model. For example, to model the working class input plus one book per day, the code randomly sampled 10 transcripts and one book 30 times and computed the average number of types for the value on day 1. On day 2, an additional 10 transcripts and a book were modelled, so on day 2 the model represented cumulative exposure to 20 transcripts and two books, and so forth over 365 days to build the cumulative exposure curve. Table 6 reports token input and cumulative type exposure for the three groups at day 365.

**Fig. 6:**
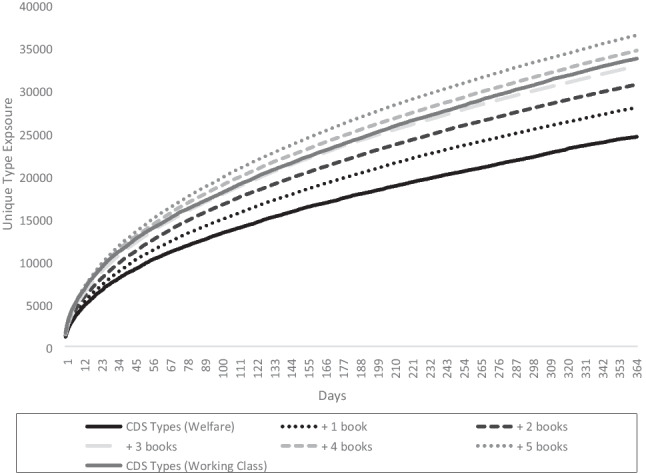
Visualisation of cumulative exposure to new types (welfare cf. working class)

Table 6 shows large lexical diversity gaps amongst the three groups when only cds types are considered. For example, the gap between welfare and working class is 17.2% at the end of the 365-day model. Montag et al. (2018) found a 16.5% increase in lexical diversity though the addition of three books per day over a simulated month. The current model is not dissimilar, indicating that at 12 months, the hypothetical welfare family child could ‘close the gap’ and have input similar in lexical diversity (types) to the working class with three books a day and surpass it at four or five. Results from days 1 to 365 are visualised in the unique type growth curves of Fig. 6, comparing the welfare input over time (solid black line) with the working class input (solid grey line) and the remaining curves representing the welfare input with the addition of 1–5 books per day.

**Fig. 7:**
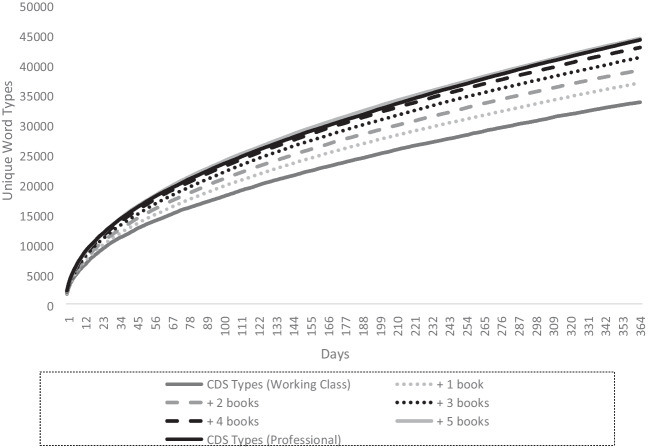
Visualisation of cumulative exposure to new types (working cf. professional class)

Figure 6 shows that one to two books per day would substantially change the curve of lexical diversity exposure. As Montag et al. (2018) noted, this model assumes new books per day. To converge on similar input to the higher SES group, 3+ new books per day are needed. This may or may not be a feasible target, but the timestamp data in cpb-lex indicate that it would only be approximately 18–30 minutes of reading per day, which does not seem unrealistic (McQuillan & Krashen, 2008). Figure 7 visualises the exposure curves comparing the working class and professional class input.

Figure 7 illustrates that a working class child’s exposure to new types can also be dramatically changed by reading picture books. However, for this group, the model required approximately four books to close the lexical diversity gap with the professional class input and five books to surpass it. Considering tokens alone, it is worth noting that Logan et al. (2019), using 60 picture books, computed that reading one book a day would add 77,896 tokens per year to a child’s input and up to 1.4 million by the time children start school if they were to read five books a day. The study concludes that regular reading could make a large contribution to closing deficit gaps. The current data support this but suggest that the contribution may be greater. In their corpus, the mean book length was 228 tokens, and they removed books with longer words counts (e.g. above 2157 words) as outliers. However, mean book length in the cpb-lex was 522 tokens. This could be due to sampling differences; for example, the books in Logan et al. (2019) may have been for younger readers than in cpb-lex. Alternatively, it may be sampling error, with 60 books not providing enough power to find that the typical token exposure children receive from picture books is potentially more than indicated in the Logan et al. (2019) model.

## Limitations

A limitation of cpb-lex is its general age guidelines, with picture books recommended for ages under 8 years (Matulka, 2008). In this sense, the resource is like cbeebies (0–6) and cbbc (6–12), rather than graded databases such as ccloww (Li et al., 2022). The above models are limited by focusing on cumulative token input and its effect on type exposure. Discussion has put aside Matthew effects, repeated readings and underlying differences in the types (Sun et al., 2019; Sun et al., 2022). Input differs in type exposure not only via its relationship to tokens, but also by its association with variables such as the size of caregivers’ vocabularies, beliefs about appropriate words for children and so on (Montag et al., 2018). A limitation of childes as the cds-proxy is that the ages of children in childes are skewed 5 years and under, while picture books are possibly for older children. This may account for some lexical diversity differences, since a cds-proxy for older children would likely have more lexical diversity than what we have modelled. Limitations pertaining to how NLP techniques work also need to be considered. Noise and error exist in POS tagging, lemmatisation, character encoding, and the ASR algorithm may have misidentified some words. Error in cpb-lex is difficult to detect as ASR captions are based on probabilities of what was in the speech stream. Rather than transcribing non-word character strings as errors that could be automatically deleted, it produces the best-matching English word, which may not be what was said. Non-text words may have been missed in manual cleaning despite the care taken. This study used original code, pre-existing tools and existing code to accomplish multiple tasks in the workflow. Programs can vary in their data handling which can result in, for example, different overall word counts for large corpora. The number of words in any (large) corpus are approximations (Sampson, 2002). Frequencies are affected by how algorithms function, such as POS tagging capturing *‘s* as a tag either as a possessive marker or contracted verb, while lemmatisation ignores the former and converts the latter (e.g., *year’s* tagged as year_NNP +_’s_POS). It is impossible to give part-of-speech or lemmatisation error rates or to manually correct computational algorithms (see Brysbaert et al., 2012, for similar arguments). Imprecision in data mining methods needs to be fully acknowledged for the research community, but we suggest that the due diligence in the methods and the results presented above speak to validation and an acceptable level of signal to noise in cpb-lex for it to constitute a highly valuable resource.

## Conclusion

Cpb-lex, a lexical database derived from children’s picture books, is a suite of resources useful for research in the behavioural sciences. Picture books are an important type of input during language and reading development. In English instruction, they are used in formal education around the world and are typically one of the most commonly used text types in emergent literacy. During child–parent interactions, picture books are thought to enrich input from child-directed speech. A database of this input is important for research in psychology, education and computer science, where rich details on vocabulary are required. This study used a data mining solution to address the challenge of obtaining lexical statistics from a large sample of children’s picture books. Our analysis indicates that lexical metrics obtained from our corpus can predict age of acquisition and can be used for purposes such as developing up-to-date lists of high-frequency words for education and testing. Cpb-lex can also be of value in computational modelling of children’s input from early print. We have shown, for example, that the vocabulary input from a relatively small number of picture books can dramatically enrich child-directed speech and potentially assist children with vocabulary input deficits. Cpb-lex has potential value to assist in research involving cognitive modelling of children’s learning. Future research could make wide use of the data for lab experiments and computational modelling research into the developmental lexicon.

## Data Availability

The datasets generated during and/or analysed during the current study are available in the Open Science Framework (OSF) repository, https://tinyurl.com/4este73c.
